# BCL2L2 loss renders ‐14q renal cancer dependent on BCL2L1 that mediates resistance to tyrosine kinase inhibitors

**DOI:** 10.1002/ctm2.348

**Published:** 2021-03-04

**Authors:** Yinfeng Lyu, Kunping Li, Yuqing Li, Hui Wen, Chenchen Feng

**Affiliations:** ^1^ Department of Urology Huashan Hospital Fudan University Shanghai P. R. China; ^2^ Institute of Urology Fudan University Shanghai P. R. China

Dear editor,

We here report BCL2L2 loss renders ‐14q clear‐cell renal cell carcinoma (ccRCC) dependent on BCL2L1 that mediates resistance to tyrosine kinase inhibitors (TKIs).

‐14q confers worsened prognosis in ccRCC but little is known about its target gene(s).^1^ We previously showed ‐14q was associated with resistance to Sunitinib (Sun) and Sorafenib (Sor).^2^, ^3^ Our findings, together with recent report that long‐recognized HIF1A may not be a ‐14q target in ccRCC,^4^ prompted us to locate candidate target(s) related to TKI resistance and worsened outcome.

Unlike deep focal deletion, shallow deletion of 14q spanned extensively and was prognostic (Figure 1A). To identify vulnerability on 14q, we designated that a candidate gene should simultaneously meet the following criteria: (a) copy number loss of the gene corresponded to substantial decrease of mRNA expression in TCGA KIRC dataset^5^; (b) loss of cytoband encompassing the gene could be mapped to at least one ccRCC cell line in COSMIC dataset and preferably but not mandatorily druggable in GDSC dataset^6^; (c) cell lines in step (b) should not be dependent on the candidate gene but be dependent on its functionally redundant counterpart in DepMap platform. We then identified BCL2L2, whose expression was significantly lower in cases with shallow deletion, indicating loss of gene function upon deletion (Figure 1B). Querying loss of cytoband 14q11.2 that encompassed BCL2L2 in GDSC pan‐cancer dataset yielded sensitivity favoring BCL inhibitor Navitoclax (Nav) (Figure 1C). When stratified to RCC cell lines only, Nav still showed significant selectivity for 14q11.2 loss (Figure 1D). As only 769P cell that harbored 14q11.2 loss was profiled in both COSMIC and DepMap platforms, we queried dependency score of BCL2L2 and its interacting components. We found BCL2L2 had highest score amid all (Figure 1E) and BCL2L1 showed lowest score of lower than ‐1, indicating dependency of 769P cell on BCL2L1. Further analysis showed strongest negative correlation between expressions of BCL2L2 and BCL2L1 (Figure 1E).

**FIGURE 1:**
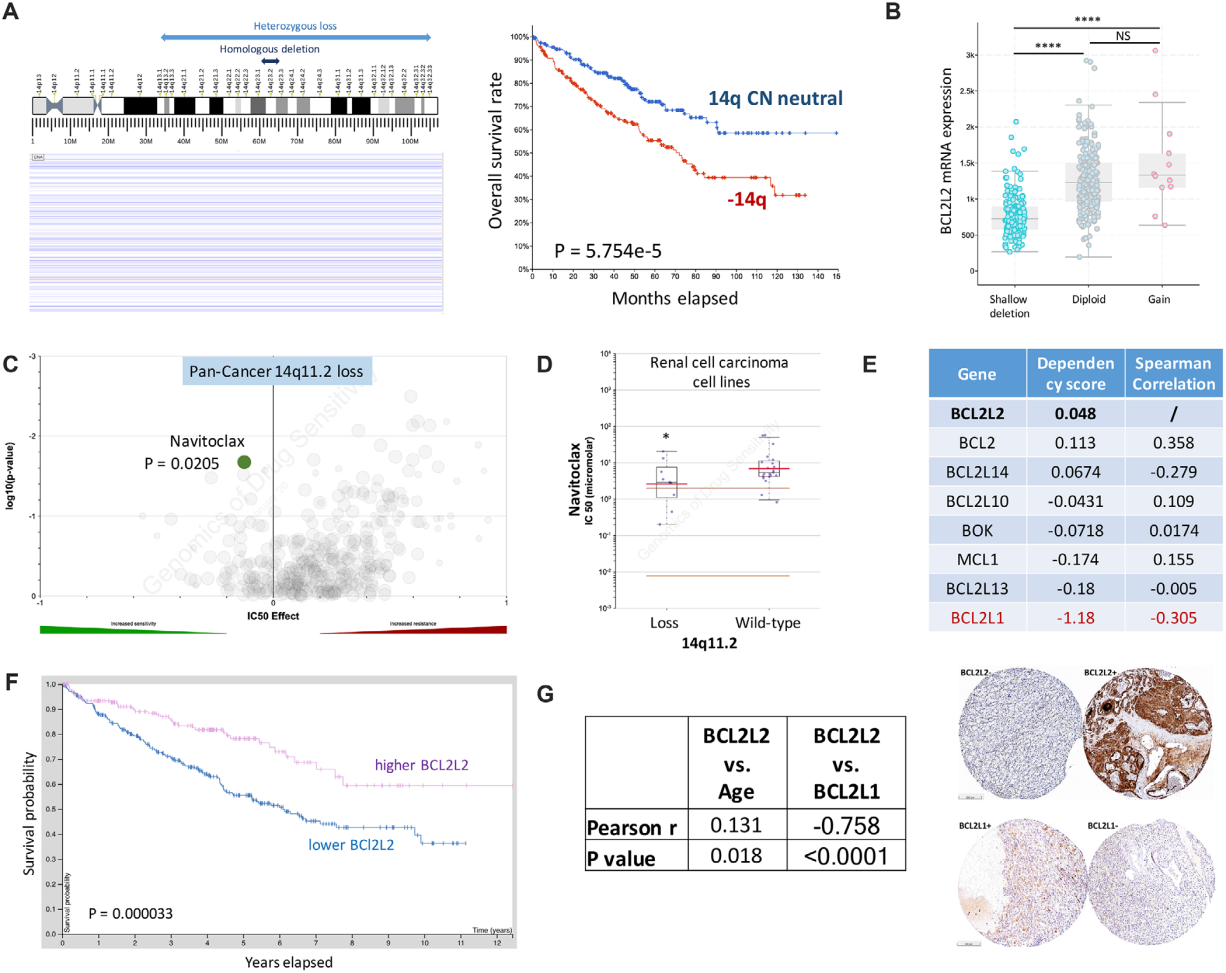
BLC2L2 could be the target gene in ‐14q ccRCC. Reproduced from the Cancer Genome Atlas clear‐cell renal cell carcinoma (ccRCC) dataset (KIRC), shown were (A) regions with shallow and deep deletion on 14q in 538 ccRCC cases and Kaplan–Meier curve showing survival plots of cases with or without 14q loss (deep and shallow); and (B) mRNA expression of BCL2L2 against its copy number in KIRC cohort. Reproduced from the Genomics of Drug Sensitivity in Cancer (GDSC) dataset, shown were (C) representative volcano plot of drug sensitivity profile for 14q11.2 loss in all cancer cells (Pan‐Cancer) with Navitoclax as candidate; and (D) sensitivity of Navitoclax in RCC cells with 14q –loss or –neutral presented with IC50. Queried from DepMap platform (https://depmap.org/portal/), shown was (E) dependency score of select BCL family genes in 769P ccRCC cells that harbored ‐14q, with lower score indicating more essentiality of the gene; Spearman correlation coefficient being retrieved from KIRC dataset showing correlation of expressions of BCL genes with that of BCl2L2. Reproduced from KIRC and generated by Human Protein Atlas platform, shown was (F) Kaplan–Meier curve showing survival plots of cases with higher or lower BCL2L2 expression with automatically designated cutoff value. (G) Embedded table representing semiquantitative immunohistochemical staining score of BCL2L2/BCL2L1 studied for correlation between age and within each other, with representative images to the left (bar = 200 μm; *P < .05; **P < .01; ***P < .001; ****P < .0001)

As the negative correlation between BCL2L1 and BCL2L2 extended to all samples with a correlation coefficient even stronger than ‐14q cases in KIRC dataset (Figure 1E) but not in any other cancer in TCGA (Figure 2A), we speculate that loss of function of BCL2L2 play a role in ccRCC. We found KIRC cases with lower BCL2L2 expression had significantly worsened prognosis (Figure 1F). For validation, we evaluated IHC score of BCL2L2 and BCL2L1 in 324 primary ccRCC samples and found BCL2L2 level was significantly lower in cases with higher stage, grade, nodal involvement, and metastasis (Table 1). Expressions of BCL2L2 and BCL2L1 also showed strong negative correlation (Figure 1G). Interestingly, both genes showed a positive correlation in normal kidney tissue (Figure 2B‐E) and differential expression was solely significant for BCL2L2 but not BCL2L1 (Figure 2F & G).

**FIGURE 2:**
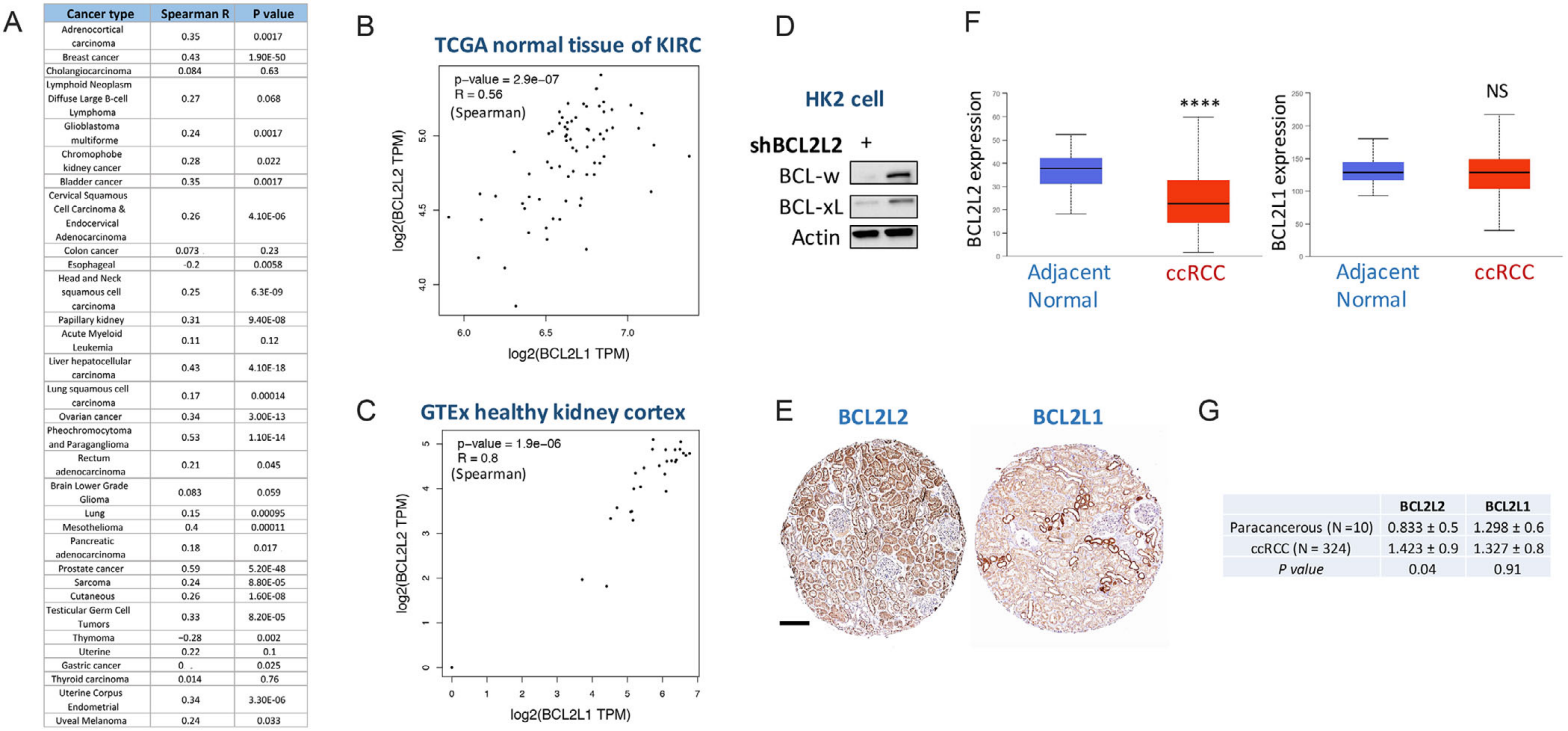
Expressions of BCL2L2 and BCL2L1 in TCGA cancers and normal kidney tissue. (A) Correlation of expressions of BCL2L2 and BCL2L1 in all TCGA cancers reproduced using the GEPIA platform. R value denotes Spearman correlation coefficient. Reproduce from (B) TCGA para‐cancerous normal kidney tissue dataset and (C) GETx healthy kidney cortex dataset, shown were Spearman correlation between BCL2L2 and BCL2L1 expression therein; (D) Expression of BCL2L2 (BCL‐w) and BCL2L1 (BCL‐xL) in normal human kidney cell line HK2; (E) representative positive immunohistochemical (IHC) staining of BCL2L2 and BCL2L1 in the same normal kidney sample; (F) Reproduce from TCGA KIRC dataset showing differential expressions of BCL2L2 and BCL2L1 in normal and cancerous tissue of renal cancer; (G) Validation of differential expression of BCL2L2 and BCL2L1 using IHC in 10 paracancerous tissue and 324 primary clear‐cell renal cell carcinoma (ccRCC) tissue presented as mean IHC score with standard deviation and compared with the *t* test (****P < .0001; NS = not significant)

**TABLE 1:** Semiquantitative immunohistochemical staining score of BCL2L2/BCL2L1 stratified by clinicopathological parameters of 324 primary ccRCC samples

	Validation				
			BCL2L2 expression		
Parameter	breakdown	N	Mean	SEM	*P*
T	T1	162	1.525	0.069	<.0001
	T2	96	1.563	0.066	
	T3	55	1.073	0.127	
	T4	10	0.400	0.267	
N	N0	274	1.522	0.050	<.0001
	N1	50	0.880	0.148	
M	M0	249	1.386	0.05503	.0005
	M1	10	0.400	0.267	
Gender	Male	196	1.449	0.062	.4994
	Female	128	1.383	0.075	
Grade	I	86	1.523	0.116	<.0001
	II	176	1.545	0.047	
	III	48	0.917	0.126	
	IV	14	1.000	0.332	
Neoadjuvant Tx	No	306	1.461	0.047	.001
	Yes	18	0.778	0.275	
			BCL2L1 Expression		
	No	306	1.301	0.046	.0183
	Yes	18	1.778	0.275	

‐14q 769P and 14q‐neutral 786O cell lines were used. Constitutive BCL2L2 (BCL‐w) was significant lower in 769P (Figure 3A & 4A). Though BCL2L1 (BCL‐xL) level was higher in BCL‐w‐low 769P cells, their levels were conversed when BCL2L2 (BCL‐w) was overexpressed (OE) in both cell lines (Figure 3A & 4A). Targeting BCL2L1 significantly inhibited proliferation of 769P cells which was in part rescued by BCL2L2‐OE. Nav not only mimicked effect of BCL2L1 but also of 786O cells with BCL2L2‐KD (Figure 3B). Nav induced significant apoptosis in BCL2L2‐KD 786O cells and in treatment‐naïve 769P cells, which could in part be restored by BCL2L2‐OE but not by MCL1, whose OE was associated with Nav resistance^7^ (Figure 3C, Supporting information Figure S1A‐B). As BCL2 and BCL‐xL were closely associated with PARP signaling,^8^ we used cleaved‐PARP to profile effect of Nav. Nav induced cleaved‐PARP in 769P cells which could be restored by BCL2L2‐OE (Figure 3D & 4B). 786O cells with BCL2L2‐KD also mimicked 769P cells by showing cleaved‐PARP upon Nav. MCL1 level remained unchanged in both cells indicating trivial participation of other BCL family proteins (Figure 3D & 4B). Nav also significantly induced increased apoptotic activity which could be restored in part by BCL2L1‐OE in 769P cells (Figure 3E). We thus hypothesized resistance to TKIs (Sunitinib and Sorafenib)^2^, ^3^ could be mediated by BCL2L2/BCL2L1 imbalance. To distinguish whether resistance rooted from BCL2L2 or gain BCL2L1, we found 14q‐neutral RCC cells (786O and A498) inherently upregulated BCL2L2 (BCL‐w) and downregulated BCL2L1 (BCL‐xL) in response to both TKIs (Figure 3F & 4C). BCL2L2‐OE overturned sensitivity in 786O cells (Figure 3G). We therefore hypothesized BCL2L1 inhibition could sensitize ‐14q RCC cells to TKIs. To avoid interference of angiogenesis, we first tested combination therapy in vitro and found synergistic inhibition in proliferation (Figure 3H). Nav (3 μM) sensitized both TKIs by reducing IC50 to 1.8 μM (Sor) and 1.4 μM (Sun), respectively, and combination induced potent apoptosis in 769P cells even when TKI dose was reduced by 30‐fold (Figure 3I, Supporting information Figure S1C‐D). Whereas Nav or low‐dose TKI alone did not alter migratory ability, the N3S1 (Nav at 3 μM and Sun or Sor at 1 μM, respectively) combination significantly decreased cell migration (Figure 3J). In xenograft models, Nav or low‐dose Sun or Sor alone only resulted in approximately 20% of tumor shrinkage whereas combination resulted in approximately 70% of size reduction (Figure 3K). In the tail‐vein injection model, only mice with combination therapy showed significantly improved survival (Figure 3L).

**FIGURE 3:**
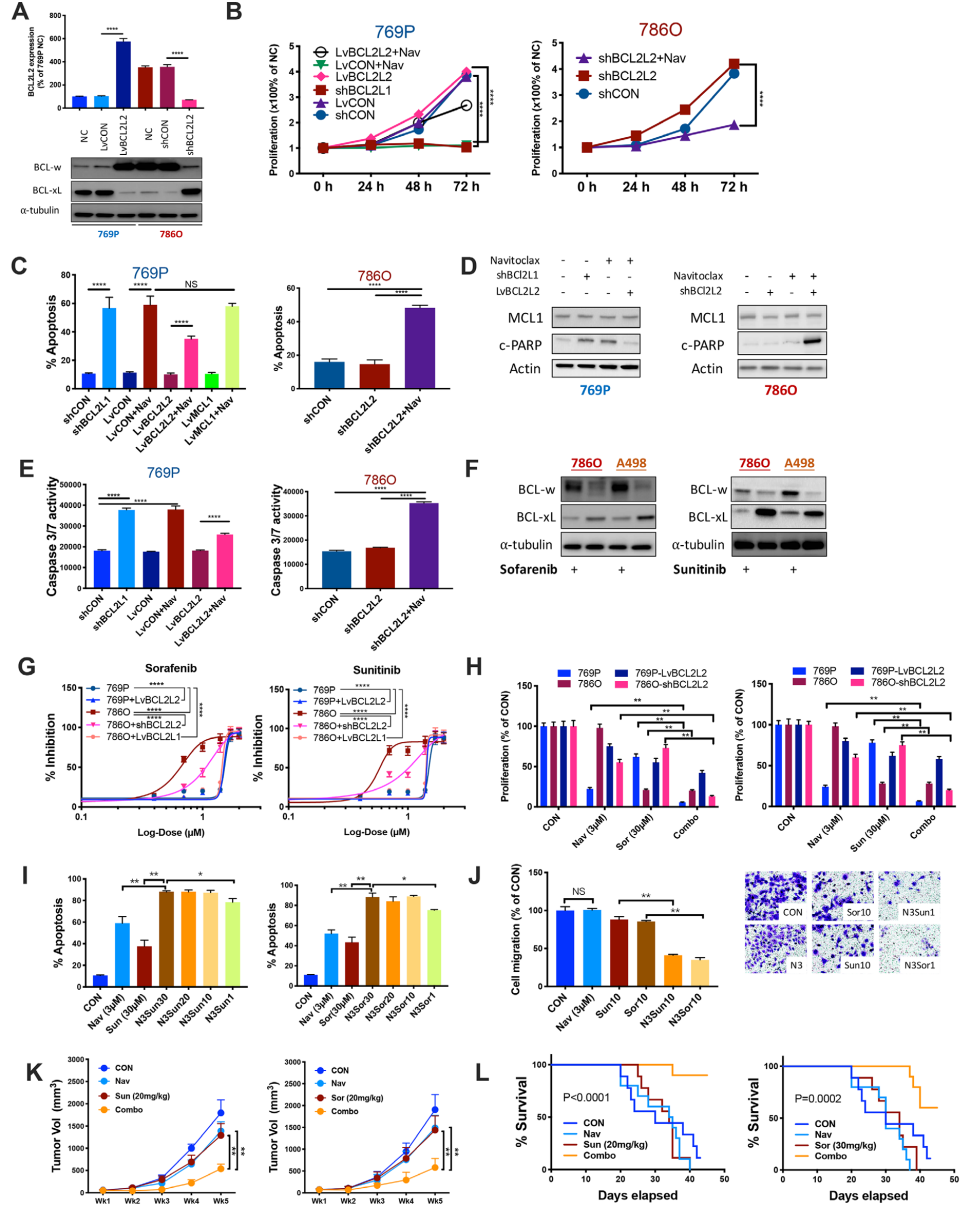
Targeting BCL2L1 inhibits ‐14q ccRCC and generates synergy with TKIs. (A) mRNA and protein level of BCL2L2 (BCL‐w) and BCL2L1 (BCL‐xL) in ‐14q ccRCC cell line 769P and 14q‐neutral 7860 cells in response to lentiviral (Lv) overexpression of and shRNA‐mediated knockdown of the genes. (B) Proliferation assay in ccRCC cell lines using crystal violet with Navitoclax (Nav) being applied at 3 μM and all data normalized to null control (NC). (C) Apoptosis assay in ccRCC cell lines using Propidium Iodide/Annexin V staining and flow cytometry with Navitoclax (Nav) being applied at 3 μM; % Apoptosis being sum of % (early + late) apoptotic cells. (D) Western blotting (WB) showing level of cleaved PARP (c‐PARP) and MCL1 in ccRCC cell lines with overexpression (Lv) or knockdown (sh) of target genes with Navitoclax (Nav) being applied at 3 μM and WB performed at 36 h post‐treatment. (E) Apoptotic activity profiled by Caspase 3/7 tested by Promega Caspase‐Glo assay in ccRCC cell lines with Navitoclax (Nav) being applied at 3 μM and WB performed at 36 h post‐treatment. (F) WB showing BCL2L2 (BCL‐w) and BCL2L1 (BCL‐xL) levels in 14q‐copy number neutral ccRCC cell lines with or without tyrosine kinase inhibitors (TKIs); both compounds were applied at dose of 10 μM for a treatment course of 24 h. (G) Shifting of sigmoidal dose‐response fitting curve of two TKIs applied to 2 ccRCC cell lines with overexpression (Lv) or knockdown (sh) of target genes. (H) Proliferation detected using crystal violet at 72 h of treatment, all normalized to control (CON). (I) Apoptosis detected by flow cytometry at 72 h of treatment with 3 μM of Navitoclax (Nav, N3) or combined with deferent doses of Sunitinib (Sun30, etc.) or Sorafenib (Sor30 etc.). (J) Transwell migration assay using crystal violet staining showing penetrated cells treatment groups of different doses of Nav and TKIs, detected at 96 h of treatment, all normalized to CON. (K) Xenograft murine models consisting of 6 BALB/c nude mice per group with subcutaneous implanted 769P cells under left flank, fed with 20 mg/kg of Sor or Sun and Navitoclax formulated in 10% ethanol, 30% polyethylene glycol 400, and 60% Phosal 50 PG (a dispersion of 50% phosphatidylcholine in a propylene glycol/ethanol carrier) orally by gavage; tumor growth monitored over 5‐week period; (L) Tail vein injection of 769P cells in 9 mice per group with Nav, Sun, Sor, or combo treatments (Tx); mice monitored for 45 days for survival (all in vitro assays performed in triplicates and at least three biological replicates; *P < .05; **P < .01; ***P < .001; ****P < .0001)

**FIGURE 4:**
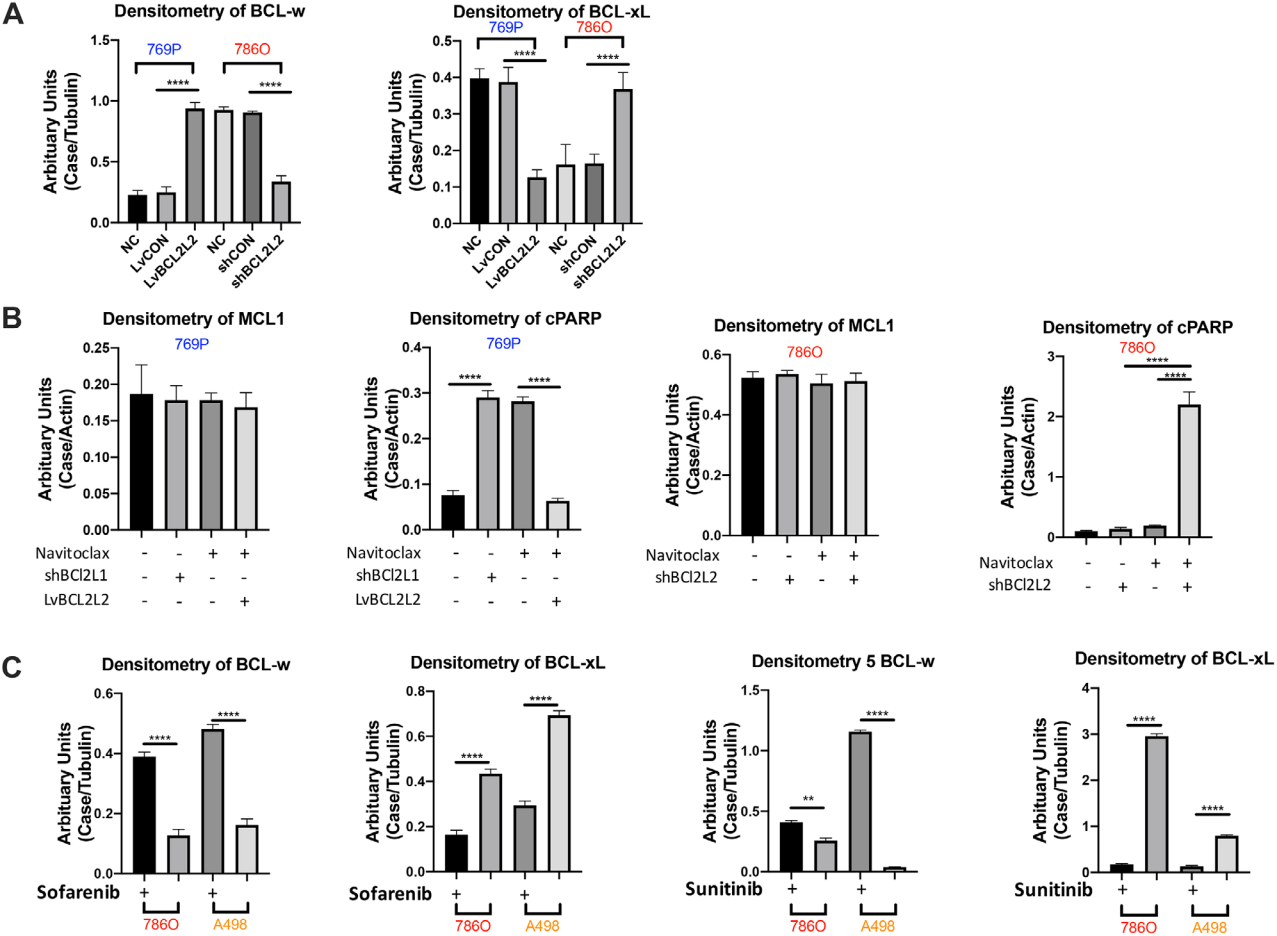
Histograms of densitometry of western blots in the current study. (A) Protein level of BCL2L2 (BCL‐w) and BCL2L1 (BCL‐xL) in ‐14q ccRCC cell line 769P and 14q‐neutral 7860 cells in response to lentiviral (Lv) overexpression of and shRNA‐mediated knockdown of the genes, corresponding to Figure 2A; (B) protein level of cleaved PARP (c‐PARP) and MCL1 in ccRCC cell lines with overexpression (Lv) or knockdown (sh) of target genes with Navitoclax (Nav) being applied at 3 μM and WB performed at 36 h post‐treatment, corresponding to Figure 2D; (C) BCL2L2 (BCL‐w) and BCL2L1 (BCL‐xL) levels in 14q‐copy number neutral ccRCC cell lines with or without tyrosine kinase inhibitors (TKIs); both compounds were applied at dose of 10 μM for a treatment course of 24 h, corresponding to Figure 2F (**P < .01; ***P < .0001)

Based on our study, we postulated that loss of BCL2L2 generated dependency on BCL2L1 to a broad extent with the strongest effect in cells with inherent loss of BCL2L2, and that ‐14q RCC tended to maintain the balance of negative correlation between BCl2L2 and BCL2L1. As TKIs exerted major cancer‐intrinsic antineoplastic effects via apoptosis induction besides antiangiogenesis in RCC^9^ and BCL‐related resistance to TKIs, including Sorafenib, has been reported in other cancers,^10^ our findings support that targeting BCL2L1 in ‐14q kidney cancer not only attacks its vulnerability, but sensitizes it to TKI as well. The findings not only showed a potential target for 14q loss in kidney cancer but also hold promise to targeted therapy for ccRCC of such genotype. Why only BCL2L1 but not BCL2L2 could mediate resistance warrants further study.

## CONFLICT OF INTEREST

The authors declare no conflict of interest.

## FUNDING

This study was sponsored in part by National Natural Science Foundation of China (Grant No. 81874123 and No. 81772709).

## ETHICS APPROVAL AND CONSENT TO PARTICIPATE

Pathological sections and metadata of deidentified patients in the current study were all from the authors’ institute, Huashan Hospital. Written consent was obtained from all patients. The protocol conformed to ethic‐waiver regulation of the Huashan Institutional Review Board (HIRB). Animals used conformed to the Fudan Laboratory Animal Ethics Board.

## AUTHORS' CONTRIBUTIONS

YLi, YLyu, KL, and CF performed experiments. HW and CF designed the study. YLyu and CF wrote the manuscript. All authors read and approved the final manuscript.

## Supporting information

Supporting information
