# Correction to: BCL2L2 loss renders ‐14q renal cancer dependent on BCL2L1 that mediates resistance to tyrosine kinase inhibitors

**DOI:** 10.1002/ctm2.1268

**Published:** 2023-05-19

**Authors:** Yinfeng Lyu, Kunping Li, Yuqing Li, Hui Wen, Chenchen Feng

**Affiliations:** ^1^ Department of Urology Huashan Hospital Fudan University Shanghai P. R. China; ^2^ Institute of Urology Fudan University Shanghai P. R. China

Following publication of the original article,^1^ the authors identified some minor errors in Figure 2F and Supplementary File, specifically:

In Figure S1B, the first apoptosis image of 786O cells, the incorrect image was used. In Figure 2F, the image of BCL‐xL in the Sorafenib group was incorrect.

The updated Figure 2 and Supplementary File is provided.

**FIGURE 2:**
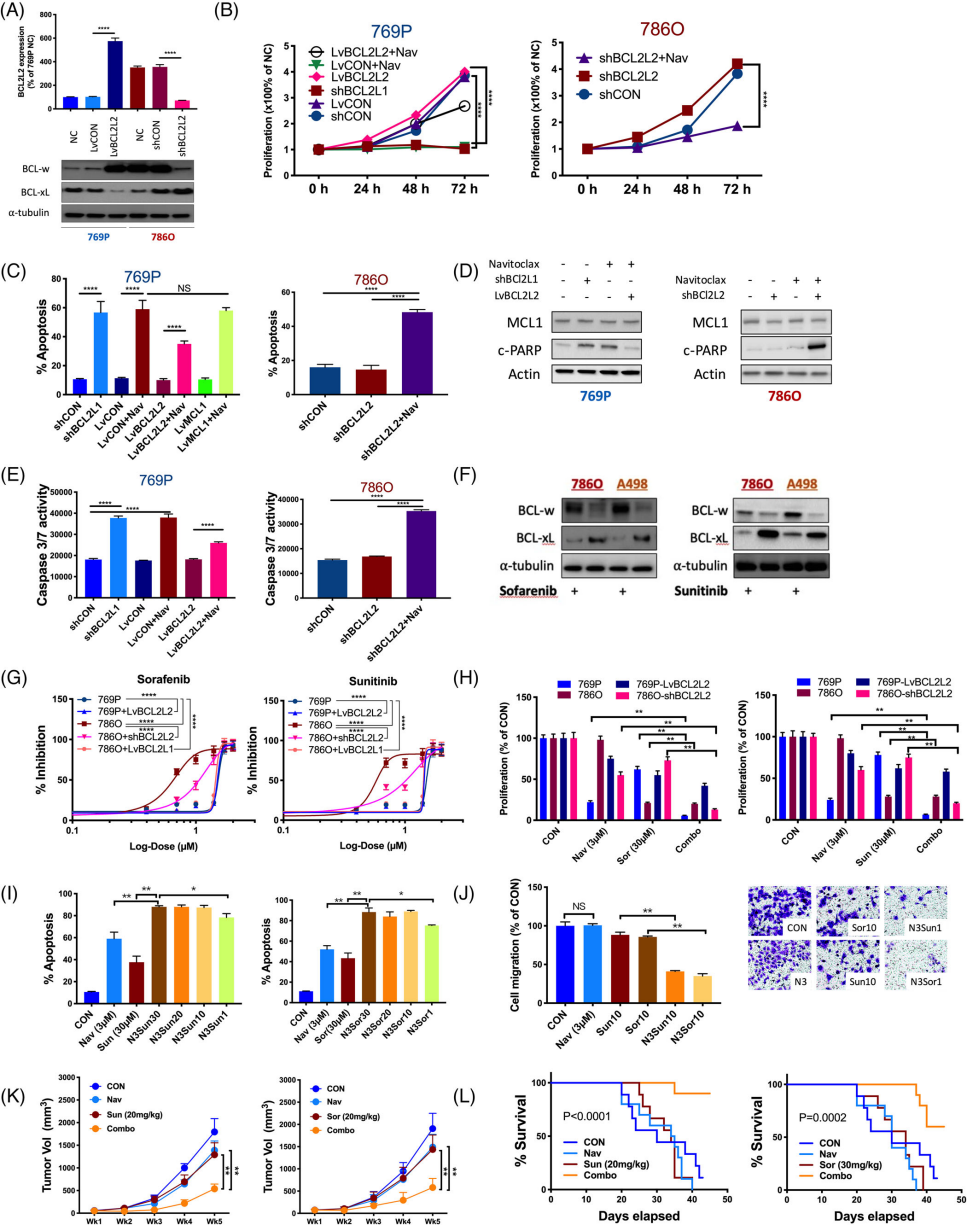


## Supporting information

Supporting Information
