# Supplementary material for: BCL2L2 loss renders ‐14q renal cancer dependent on BCL2L1 that mediates resistance to tyrosine kinase inhibitors
Source: Clin Transl Med. 2021 Mar 4;11(3):e348. doi: 10.1002/ctm2.348 (PMC7933017; doi:10.1002/ctm2.348)
Supplement: Supplementary file 1 — Supporting information [file CTM2-11-e348-s001.pdf]

SUPPLEMENTARY FILES

BCL2L2 loss renders -14q renal cancer dependent on BCL2L1 that mediates resistance to tyrosine kinase inhibitors

Running title: BCL2L2 as TKI-resistance target on 14q in renal caner

Yinfeng Lyu[1,2#], Kunping Li[1,2#], Yuqing Li[1,2#], Hui Wen[1,2*], and Chenchen Feng[1,2*]

[1]Department of Urology, Huashan Hospital; [2]Institute of Urology; Fudan University, Shanghai 200040, PR China

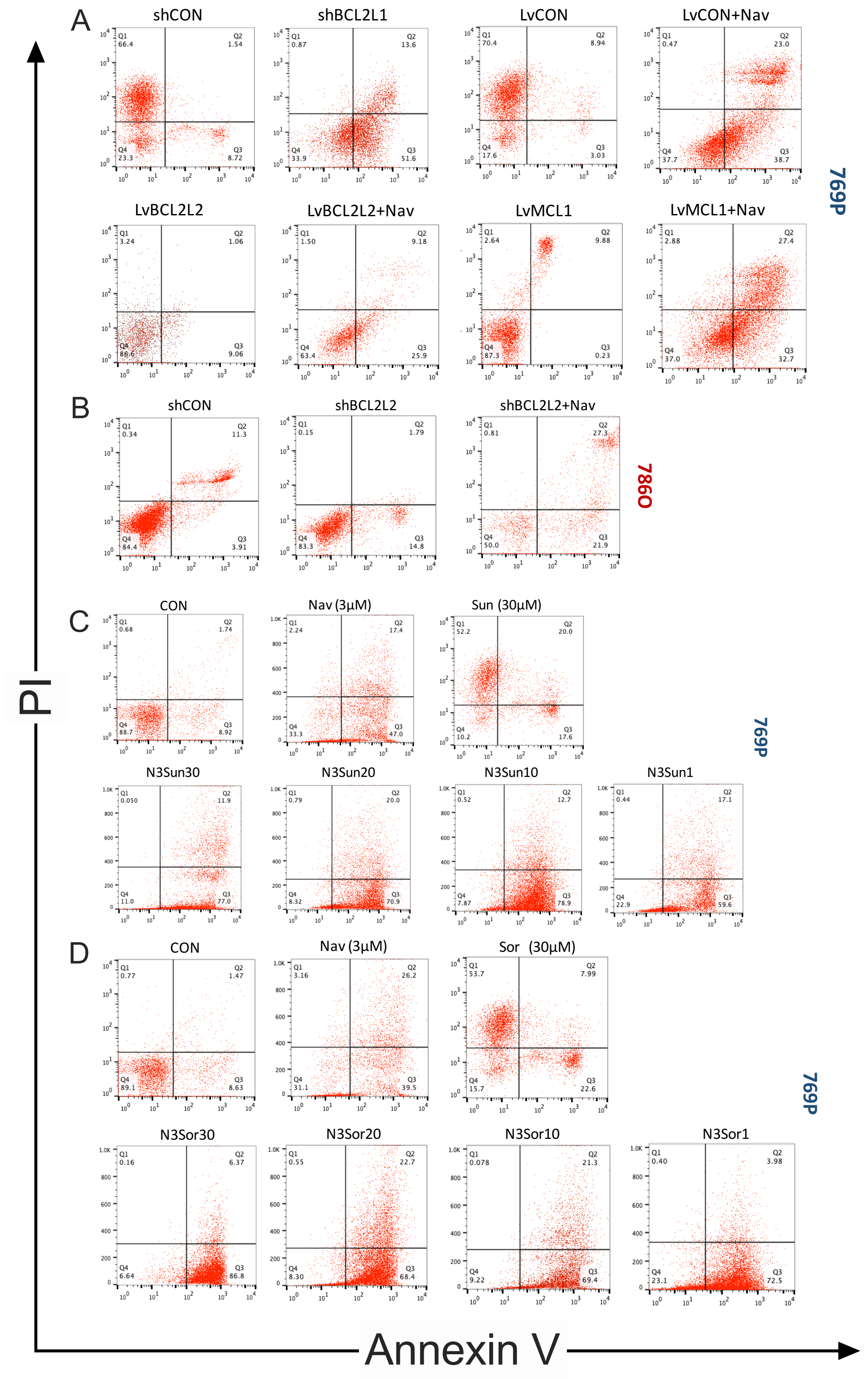

Supplementary figure 1. Flow cytometry using double staining of Propidium Iodide (PI) and Annexin V to detect apoptosis in the current study. **A -B)** Apoptosis assay in ccRCC cell lines with Navitoclax (Nav) being applied at 3 μM; % Apoptosis being sum of % (early + late) apoptotic cells, corresponding to Figure 2C; **C-D)** Apoptosis detected by flow cytometry at 72 h of treatment with 3 μM of Navitoclax (Nav, N3) or combined with deferent doses of Sunitinib (Sun30, etc.) or Sorafenib (Sor30 etc.), corresponding to Figure 2I.

Supplementary methods

In silico analysis

Reproduction of TCGA KIRC dataset was performed on the cBioPortal online platform (http://www.cbioportal.org/) with the selection of Firehose Legacy subsets (1, 2). Copy number variance (CNV) dataset was used with GISTIC value of <-2 designated as deep deletion and between -1 and -2 designated as shallow deletion. BCL2L2 (B-cell lymphoma 2 like 2) CNV was queried in KIRC dataset against mRNA expression in tumor tissues and were plotted with cBioPortal. Dependency score of mRNA expression in ccRCC (clear-cell renal cell carcinoma) cell lines were analyzed and with DepMap Portal online platform (https://depmap.org/portal/), which integrated genomic data from various high throughput sequence resources. Survival analysis for was performed at Human Protein Atlas platform (https://www.proteinatlas.org/). Expression cutoff for BCL2L2 (high vs. low) was automatically designated by the platform in the survival analysis. The Harmonizome platform was used to examine copy number of BCL2L2 in OV cell lines (http://amp.pharm.mssm.edu/Harmonizome/). The GDSC (Genomics of Drug Sensitivity in Cancer) dataset (https://www.cancerrxgene.org/) (3)was used for drug sensitivity screening targeting 14q11.2 loss (cnaPANCAN397) in pan-cancer cell lines and renal cancer cell lines.

Tissue microarray (TMA) and immunohistochemistry (IHC)

TMA sections of ccRCC and adjacent normal tissues were collected from tissue bank of Huashan Hospital. Section was de-identified and only information on cancer subtype, TNM stage, tumor grade and demographic information were available. A standard IHC protocol was followed. Briefly, section was deparaffinized after incubation for 30 min. Before graded dehydration, section was immersed in xylene for 15 min and 1:1 of xylene and alcohol for 10 min. 3% of hydrogen peroxide was used for blockade at room temperature. Antigen recovery was prepared in 0.01M sodium citrate buffer solution (pH 6.0) in a microwave for 20 min. After cooling, 10% serum in TBS was used for blockade. The primary antibody for BCL2L2 (Abcam, ab190952, at 1:100) and BCL2L1 (Abcam, ab270253, at 1:100) were applied overnight. After rinsing twice with TBS, secondary antibody was applied. After rinsing 4 times, section was stained with Vulcan Fast Red Chromogen kit, then with DAB and subsequently with hematoxylin. Sections were finalized by graded dehydration and were mounted for observation. IHC scoring for each sample was the product of intensity and extensity of the immunopositive cells (4).

Cell culture and Treatment

769P, 786O and A498 ccRCC cancer cells were obtained from CellScource China. Cells were cultured in RPMI-1640 medium supplemented with 10% of FBS.The GPP Web Portal (https://portals.broadinstitute.org/gpp/public/) was used for shRNA construction targeting BCL2L2 and BCL2L1. cDNA clone for BCL2L2, BCL2L1 and MCL1 were obtained from Origene. Overexpression was realized by lentiviral delivery using polybrene system. Quantitative PCR was performed to examine the shRNA effect and constitutive BCL2L2/BCL2L1 expression level in different ccRCC cell lines. Primers were constructed using the PrimerBank (https://pga.mgh.harvard.edu/primerbank/). Treatment of Sorafenib, Sunitinib and Navitoclax were respectively indicated in figure legends of different assays.

Western blotting

A standard protocol of western blotting was performed. Briefly, Cells were lysed using RIPA buffer and total protein was extracted. After concentration was determined, protein was loaded with buffer onto SDS-P AGE gel with subsequent electrophoresis.

Protein was then transferred to PVDF membrane which was blocked with 5% non-fat milk. The primary antibody for BCL2L2 (BCL-w) (Abcam, ab190952, at 1:200), BCL2L1 (BCL-xL) (Abcam, ab270253, at 1:200), MCL1 (Abcam, ab32087, at 1:500), and PARP (Abcam, ab191217, at 1:500) were applied overnight. Corresponding secondary antibody and ECL were routinely applied. Densitometry was analyzed using ImageStudio software.

Proliferation assay

Cell proliferation was studied using crystal violet (CV) assay. Briefly, cells seeded in 96-well plates were stained using crystal violet at set time points. Cells were than treated with methanol and rinsed for excessive CV and plates were read on a plate reader.

Flow cytometry

The FASCanto flow cytometry system was used to measure apoptosis, cells were applied with Annexin V and PI and apoptotic cells were defined as sum of early and late apoptotic cells.

Caspase assay

Established protocol was followed as per Promega Caspase-Glo kit. Cells were seeded in 96-well plate and cultured for 3 days. Cells were resuspended at 1×10^4 cells/ well and 100 µl of pre-mixed Caspase-Glo reaction fluid was added. After gentle shaking, cells were subject to a plate reader.

Colony formation

72 h after viral infection, approximately 400-1000 cells were seeded in each well of a 6-well plate. Medium was changed every 3 days. Cells were fixed with 4% methanol on day 11 and subsequently stained by crystal violet.

Transwell assays

The migration were measured by Transwell assay. Cells were seeded in the upper chamber of the Transwell plate at the density of 1×10^6/ml uncoated with Matrigel. Upper chamber was supplemented with serum-free media whilst the lower chamber was filled with complete medium. Cells that penetrated were stained with crystal violet and counted for number.

In vivo study

Xenograft mouse model was performed with subcutaneous (s.c.) tumor implantation. Approximately 10^7 769P cells were injected s.c. at axillary region of 6 male mice at 4 weeks of age per group. Mice were fed with 20mg/kg of Sor or Sun and Navitoclax formulated in 10% ethanol, 30% polyethylene glycol 400, and 60% Phosal 50 PG (a dispersion of 50% phosphatidylcholine in a propylene glycol/ethanol carrier) orally by gavage. Tumors were calibrated every 3 days and mice were euthanized on the fifth checkpoint unless tumors reached 2000mm^3 of size calibrated using the formula Length * Width^2 * 0.523. Survival was monitored in tail-vein injection model. Tail vein injection of 769P cells in 9 mice per group with Nav, Sun, Sor or combo treatments (Tx); mice monitored for 45 days for survival.

Statistical analysis

Statistical analysis for in silico studies were automatically performed with the platforms used, as aforementioned. Statistical analysis for in vitro assays and in vivo experiments were performed using the Prism Graphpad 9.0 for Mac. All assays were performed in triplicates. Comparisons between two groups were studied using the Mann-Whitney test for non-parametric variants and using the Student's t test for parametric variants. IC50 for drug treatment was interpolated and fitted with sigmoidal curve. The survival data was presented using the Kaplan-Meier curve and compared using the Log-rank test. The P value of < .05 was accepted as significant.

References

1. Gao J, Aksoy BA, Dogrusoz U, Dresdner G, Gross B, Sumer SO, et al. Integrative Analysis of Complex Cancer Genomics and Clinical Profiles Using the cBioPortal. Science Signaling. 2013;6(269):pl1-pl.
2. Cerami E, Gao J, Dogrusoz U, Gross BE, Sumer SO, Aksoy BA, et al. The cBio Cancer Genomics Portal: An Open Platform for Exploring Multidimensional Cancer Genomics Data: Figure 1. Cancer Discovery. 2012;2(5):401-4.
3. Yang W, Soares J, Greninger P, Edelman EJ, Lightfoot H, Forbes S, et al. Genomics of Drug Sensitivity in Cancer (GDSC): a resource for therapeutic biomarker discovery in cancer cells. Nucleic Acids Res. 2013;41(Database issue):D955-61.
4. Feng C, Guan M, Ding Q, Zhang Y, Jiang H, Wen H, et al. Expression of pigment epithelium-derived factor in bladder tumour is correlated with interleukin-8 yet not with interleukin-1α. Journal of Huazhong University of Science and Technology--Medical Sciences--. 2011;31(1):21-5.
